# Phase I clinical trial and pharmacokinetic evaluation of NK911, a micelle-encapsulated doxorubicin

**DOI:** 10.1038/sj.bjc.6602204

**Published:** 2004-10-12

**Authors:** Y Matsumura, T Hamaguchi, T Ura, K Muro, Y Yamada, Y Shimada, K Shirao, T Okusaka, H Ueno, M Ikeda, N Watanabe

**Affiliations:** 1 Investigative Treatment Division, National Cancer Center Research Institute East, 6-5-1 Kashiwanoha, Kashiwa 277-8577, Japan; 2 Department of Medicine, National Cancer Center Hospital, 5-1-1 Tsukiji, Tokyo 104-0045, Japan; 3 Pharmacokinetics Group, Technical Development Department, Nippon Kayaku Co. Ltd, 3-31-12 Shimo, Kita-ku, Tokyo, Japan

**Keywords:** DDS, pharmacokinetics, NK911, micelle, doxorubicin, EPR effect

## Abstract

NK911 is a novel supramolecular nanocarrier designed for the enhanced delivery of doxorubicin (DXR) and is one of the successful polymer micelle systems to exhibit an efficient accumulation in solid tumours in mice. The purpose of this study was to define the maximum-tolerated dose (MTD) and dose-limiting toxicities (DLTs) of NK911 and to evaluate its pharmacokinetic profile in man. NK911 was given intravenously to patients with solid tumours every 3 weeks using an infusion pump at a rate of 10 mg DXR equivalent min^−1^. The starting dose was 6 mg DXR equivalent m^−2^, and the dose was escalated according to the accelerated titration method. A total of 23 patients participated in this study. Neutropenia was the predominant haematological toxicity, and grade 3 or 4 neutropenia was observed at doses of 50 and 67 mg m^−2^. Common nonhaematological toxicities were mild alopecia, stomatitis, and anorexia. In the dose identification part of the study, DLTs were observed at a dose of 67 mg m^−2^ (grade 4 neutropenia lasting more than 5 days). Thus, this dosage level was determined to be the MTD. Infusion-related reactions were not observed in any cases. The *C*_5 min_ and area under the concentration curve parameters of NK911 exhibited dose-dependent characteristics. Among the 23 patients, a partial response was obtained in one patient with metastatic pancreatic cancer. NK911 was well tolerated and produced only moderate nausea and vomiting at myelosuppressive dosages. The recommended phase II dose was determined to be 50 mg m^−2^ every 3 weeks.

Agents categorised as drug delivery systems (DDSs) have been developed based on the characteristic macroscopic features of solid tumours, such as hypervascularity, an irregular vascular architecture, the presence of several vascular permeability factors stimulating extravasation within the cancer, and the relatively poor drainage of macromolecules and particulates from cancer tissue. These characteristics, which are unique to solid tumours, constitute the basis of the enhanced permeability and retention (EPR) effect (Matsumura and Maeda, 1986; Maeda *et al*, 2000). Macromolecules have long plasma half-lives because they are too large to pass through normal vessel walls unless they are trapped by the reticuloendothelial system (RES) in the cells of various organs. Such macromolecular agents can diffuse out of tumour blood vessels, reach the solid tumour tissue, and be retained for a long period because of the EPR effect.

To maximise the EPR effect, several techniques have been developed to modify the structures of drugs and to construct drug carriers. Doxil is comprised of doxorubicin (DXR) encapsulated in STEALTH™ liposomes, which are composed of a phospholipid bilayer with surface-bound methoxypolyethyleneglycol. Doxil recently received the US Food and Drug Administration’s (FDA) approval for use in the treatment of Kaposi sarcoma or ovarian cancer after the clinical benefits of this drug were clearly shown in recent clinical trials (Muggia *et al*, 1996; Stewart *et al*, 1998; Gordon *et al*, 2001).

Polymeric micelles have also been utilised as a drug carrier system. The original form of micellar DXR contained two trapped components: a DXR monomer and a DXR dimer in the inner core (Yokoyama *et al*, 1990a, 1990b). However, the lyophilised micelle containing DXR dimers became insoluble after long periods of storage. To improve the solubility of this drug carrier system, a new type of polymeric micelle containing only the DXR monomer, known as NK911, has been developed (Nakanishi *et al*, 2001). The DXR monomers, rather than the DXR dimers, were thought to play a major role in the antitumour activity of the original micellar DXR drug preparation. The DXR dimers, on the other hand, were thought to stabilise the drug’s conformation. Thus, NK911, which only contains DXR monomers, is less stable in aqueous media than the original form of micellar DXR (Nakanishi *et al*, 2001; Tsukioka *et al*, 2002).

Both polyethyleneglycol (PEG)-liposomal and micellar DXR have longer plasma half-lives, accumulate in tumours more effectively because of the EPR effect, and exhibit a stronger antitumour activity than free DXR when administered in mice. Both the plasma area under the concentration–time curve (AUC) and the tumour AUC of NK911 are, however, lower than those of doxil because NK911 is less stable in the bloodstream than doxil (Working *et al*, 1994; Nakanishi *et al*, 2001). At this stage, however, we have no definitive idea as to which formulation exerts a superior antitumour activity *in vivo* because an evaluation of the activity *in vivo* cannot be based solely on the enhanced tumour AUC; several additional factors, including the efficiency of the free drug inside the formulation and the distribution of the free drug throughout the tumour tissue, must also be taken into consideration. Proper selection of the type of DDS formulation is likely to depend on the tumour vessel density of the tumour tissue. With this in mind, we conducted a phase I clinical trial on the use of NK911 in patients with solid tumours. The study was performed at the National Cancer Center Hospital, Tokyo, Japan. Our objectives were to assess the safety and toxicity profile of NK911 and to determine the maximum-tolerated dose (MTD), the phase II recommended dose, and the pharmacokinetics of NK911 in man.

## PATIENTS AND METHODS

The study protocol was reviewed and approved by the Institutional Review Board of the National Cancer Center, Tokyo.

### Therapeutic agent

Figure 1

**Figure 1:**
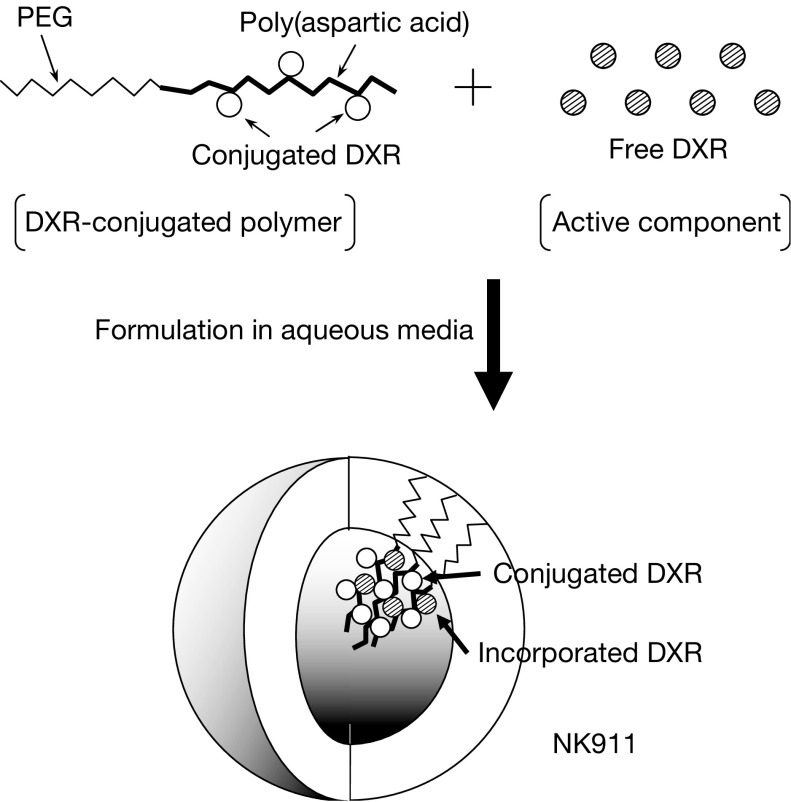
Schematic structure of NK911. A polymeric micelle carrier of NK911 consists of a block copolymer of PEG (molecular weight of about 5000) and poly(aspartic acid) (about 30 units). Polyethyleneglycol is believed to be the outer shell of the micelle. NK911 has a highly hydrophobic inner core, and therefore can entrap sufficient amounts of DXR.

shows the schematic structure of NK911, a DXR-entrapped polymeric micelle formulation. The polymeric micelle consists of a PEG–poly(aspartic acid) block copolymer conjugated with DXR. Polyethyleneglycol is believed to form the outer shell of the micelle, producing a ‘stealth’ effect that prevents NK911 from being captured by the RES. The DXR-conjugated poly(aspartic acid) chain is hydrophobic and is believed to form the hydrophobic inner core of the micelles in aqueous media. The hydrophobic inner core enables NK911 to entrap a sufficient amount of DXR. NK911 has a diameter of about 40 nm (Nakanishi *et al*, 2001).

### Patients

Patients with metastatic or recurrent solid tumours refractory to conventional chemotherapy and for whom no effective therapy was available were eligible for enrolment in this study, provided that the following criteria were met: a histologically confirmed malignant tumour; a performance status of ⩽2; an age of ⩾20 and <75 years; a normal haematological profile (neutrophil count ⩾2000 *μ*l^−1^, platelet count ⩾100 000 *μ*l^−1^, haemoglobin ⩾8 g dl^−1^); normal hepatic function (total bilirubin level ⩽1.5 mg dl^−1^, aspartate aminotransferase (AST) and alanine aminotransferase (ALT) ⩽2.5 times the upper normal limit); normal renal function (serum creatinine ⩽1.5 mg dl^−1^); normal cardiac function (New York Heart Association (NYHA) classification of ⩽1); normal pulmonary function (PaO_2_ ⩾60 mgHg); no chemotherapy within 4 weeks (6 weeks for nitrosourea or mitomycin C chemotherapy) of the administration of NK911; and a life expectancy of more than 2 months. Patients with any serious infection (including hepatitis B, hepatitis C, or HIV), symptomatic brain metastasis, and pre-existing cardiac disease (including congestive heart failure, myocardial infarction, or angina pectoris within 3 months) were ineligible for enrolment in the study. Patients were also excluded if they were pregnant or lactating or showed signs of gastrointestinal bleeding. Additionally, any patients whom the investigators considered ineligible were excluded. Written informed consent was obtained from all patients.

### Drug administration

NK911 was dissolved in sterile phosphate-buffered saline for injection at room temperature at a concentration of 2 mg DXR equivalent ml^−1^. NK911 solution was infused intravenously using an electric pump at a speed of 10 mg DXR equivalent min^−1^.

### Dosage and dose escalation

The starting dosage of NK911 was 6 mg DXR equivalent, which is one-tenth of the LD_10_ in rats. NK911 was administered once every 3 weeks, and the treatment was continued unless a severe adverse event or disease progression was observed. Dose escalation was performed according to the previously described accelerated titration method (Simon *et al*, 1997). Toxicity was graded from 1 to 4 using the NCI common toxicity criteria (version 12). Intrapatient dose escalation was not permitted. The dose-limiting toxicity (DLT) was confirmed in at least six out of 23 patients. The MTD was defined as the level at which three out of six patients experienced a DLT. The recommended dosage for a phase II trial was defined by the Efficacy and Safety Assessment Committee, based on the safety and efficacy results of this trial. The DLT was defined as grade 4 neutropenia lasting more than 5 days or an associated neutropenic fever of more than 38.5°C, a platelet count of less than 25 000 *μ*l^−1^, or grade 3 or higher nonhaematological toxicity, with the exception of nausea, vomiting, appetite loss, constipation, and hyperglycaemia.

### Pretreatment assessment and follow-up care

A complete medical history and physical examination, performance status evolution, complete blood cell count (CBC), blood chemistry, urinalysis, electrocardiogram (ECG), and a computed tomography (CT) examination or an upper gastrointestinal (GI) series were performed in each patient. Other examinations were performed only in the presence of a specific clinical indication. Patients were physically examined everyday until the second administration of NK911; CBC and blood chemistry tests were performed on days 1, 2, and 4 and weekly thereafter. Electrocardiogram and ultrasonic cardiography studies were repeated prior to each administration of NK911. Tumour markers were also measured prior to each administration. Tumour response was evaluated according to the WHO guidelines (World Health Organization, 1979). A complete response (CR) was defined as the disappearance of the cancerous lesion(s), and a partial response (PR) was defined as a reduction of more than 50% of the sum of the bidimensional products when the results for two observations, separated by at least 4 weeks, were compared. Stable disease (SD) was defined as a reduction of less than 50% or a growth of less than 25% over a period of at least 4 weeks. Progressive disease (PD) was defined as a growth of more than 25%, the appearance of new malignant lesion(s), or the unequivocal worsening of other clinical evidence of malignancy.

### High-performance liquid chromatography determination of DXR and its metabolites

Doxorubicin and its metabolites (doxorubicinol and aglycones) were extracted from human plasma and urine using Abselut NEXUS cartridges (Varian), pretreated with methanol (2 ml), 1% phosphoric acid (1 ml), and sodium phosphate buffer (2 ml of 25 mmol l^−1^ NaH_2_PO_4_ (pH 4) containing 0.2% sodium lauryl sulphate). Daunorubicin was added to the plasma (0.5 ml) or urine (0.1 ml) samples as an internal standard. The diluted plasma or urine was applied to the above-mentioned cartridges and then washed with distilled water (1 ml) or 40% methanol (2 ml) and eluted with methanol (2 ml). The eluate was evaporated to dryness at 40°C under a stream of nitrogen. The residue was dissolved in sodium phosphate buffer (200 *μ*l of 25 mmol l^−1^ NaH_2_PO_4_ (pH 4) containing 0.2% sodium lauryl sulphate/acetonitrile=75/25, v v^−1^), and 50 *μ*l of the resulting solution was injected onto the analytical column. The analyses of DXR and its metabolites in plasma and urine samples were performed using high-performance liquid chromatography (HPLC) with fluorescence detection. The DXR concentrations determined in the present Phase I study represented the total drug concentrations (both micelle-entrapped and nonencapsulated). The HPLC system (HP1100 series, Hewlett Packard) consisted of a binary pump, an automatic sample injector, a reversed-phase CAPCELL PAK C_8_ (2.0 mm i.d. × 150 mm, 5 *μ*m, SHISEIDO), and a fluorescence detector with excitation and emission wavelengths of 500 and 550 nm, respectively. A gradient elution was employed, consisting of 25 mmol l^−1^ NaH_2_PO_4_ (pH 4) containing 0.2% sodium lauryl sulphate/acetonitrile at ratios of 75/25 to 55/45 (v v^−1^), at a flow rate of 0.2 ml min^−1^.

### Pharmacokinetic analysis

The pharmacokinetic parameters were calculated after fitting the data to a three-compartment model using the Win Nonlin program. The plasma DXR concentration, *C*(*t*), at each time (*t*), was computed using the equation


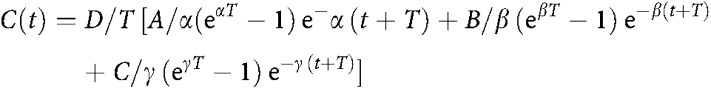


where *D* is the dose and *T* is the infusion time.

The AUC, total clearance (CL_tot_), volume of distribution at steady state (*V*_ss_), area under the first moment curve (AUMC), volume of distribution of the central compartment (*V*1), half-lives (*t*_1/2*α*_, *t*_1/2*β*_, *t*_1/2*γ*_), and mean residence time (MRT) of DXR were calculated using the equations listed below.


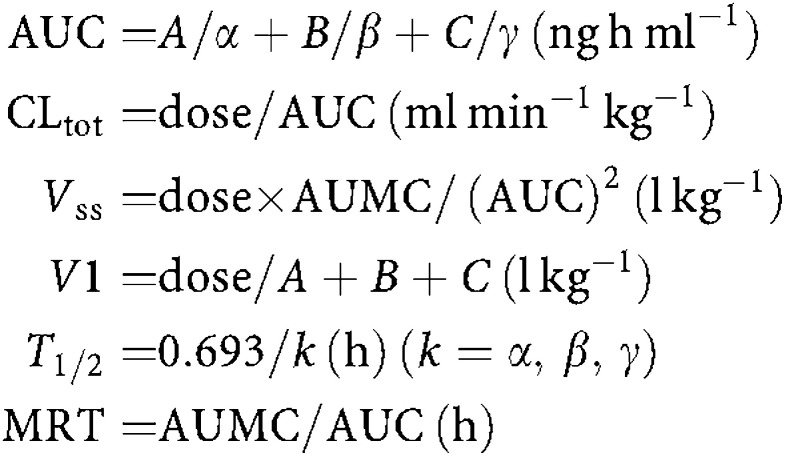


## RESULTS

### Patient characteristics

A total of 23 eligible patients were recruited for the study. Their clinical characteristics are shown in Table 1

**Table 1:** Patient characteristics

Number of patients	23
Male/female	15/8
Age (years)	
Median	61.5
Range	48–72
ECOG PS	
Median	1
0	6
1	17
Prior treatment chemotherapy regimens	
Median	2
Range	0–6

. With the exception of one patient with a leiomyosarcoma, all the patients had received chemotherapy prior to enrolment in the study. Prior therapies ranged from 0 to 6 regimens of chemotherapy. None of the patients had received anthacycline chemotherapy. As a deviation from the ordinary phase I trial protocol, patients with metastatic pancreatic cancer were deliberately recruited, based on the previously described characteristics of NK911. All patients were included in the safety and response analyses.

### Dosing

Dosage escalation started at 6.0 mg DXR equivalent m^−2^ and was increased up to 67 mg DXR equivalent m^−2^. Infusion time ranged from 58 s to 12 min and 15 s depending on the absolute dosage of NK911. In total, 63 administrations were performed in 23 patients. A total of 14 patients received more than two administrations. The maximum number of treatments was 10 courses at level 3; the average number of administrations at all levels was 2.7 courses. Up until the third level, grade 2 toxicity was not observed during the first course of chemotherapy. According to the original protocol, the dosage of NK911 should have been doubled for each escalation. However, the safety committee recommended that the dosage should be raised by 50%, instead of 100%, at level 4 and that a modified Fibonacci escalation method should be implemented. Therefore, we recruited three patients at this dosage level and restarted the dose identification study using a modified Fibonacci method.

### Haematological toxicity

As shown in Table 2

**Table 2:** Haematological toxicity: cycle 1

		**Leucocytopenia**				**Neutropenia**				**Thrombocytopenia**			
		**Grade**				**Grade**				**Grade**			
**Dose level (mg m^−2^)**	***n***	**1**	**2**	**3**	**4**	**1**	**2**	**3**	**4**	**1**	**2**	**3**	**4**
6	1	0	0	0	0	0	0	0	0	0	0	0	0
12	1	0	0	0	0	0	0	0	0	0	0	0	0
24	1	1	0	0	0	1	0	0	0	0	0	0	0
36	3	1	0	0	0	0	1	0	0	0	0	0	0
50	11	3	5	3	0	1	3	5	2	2	0	0	0
67	6	0	1	5	0	0	0	0	6	3	0	1	0
													
Total	23	5	6	8	0	2	4	5	8	5	0	1	0

, significant myelosuppression was not observed up to level 4. At level 5, two patients died because of tumour progression. The progression of the disease was confirmed by autopsy in both cases. Since these two patients could not be assessed for safety, an additional two patients were enrolled at level 5. Two patients developed grade 3 neutropenia and one patient developed grade 4 neutropenia. However, none of the patients developed a DLT at this dosage level. At level 6, all six patients who entered at this level developed grade 4 neutropenia; three of the six patients appeared to have acquired a DLT (grade 4 neutropenia lasting for more than 5 days). Based on these results, level 6 was considered to be the MTD, with neutropenia as the DLT. Since a dosage of 50 mg m^−2^ was considered to be the recommended dosage for phase II studies, an additional six patients were enrolled at a dosage of 50 mg m^−2^; one of these six patients developed a DLT in the form of febrile grade 4 neutropenia.

### Nonhaematological toxicity

The NK911 injection was generally uneventful and well tolerated. The major nonhaematological toxicities were nausea, vomiting, and anorexia. All of these toxicities were controllable. Severe mucositis and skin toxicity in the form of hand-foot syndrome did not occur. Alopecia was also mild, and only three patients experienced grade 2 alopecia after repeated doses of NK911 at levels 5 and 6 (Table 3

**Table 3:** Nonhaematological toxicity

	**Grade**				
	**1**	**2**	**3**	**4**	**Total**
Nausea	10	2	3	0	15
Vomiting	5	2	1	0	8
Anorexia	11	3	3	0	17
Fatigue	1	1	0	0	2
Stomatitis	5	0	0	0	5
Alopecia	12	0	—	—	12

There was no DLT regarding nonhaematological toxicities within this trial.

). A few patients at level 5 or 6 developed a grade 2 elevation in AST or ALT, but these changes were transient. No pain or local toxicity in the area of injection was observed in any of the patients treated with NK911, except in one patient treated at level 2. No infusion-related reactions were observed in any cases; such reactions sometimes occur during liposomal drug administration. Cardiac function was monitored at baseline and serially in all patients enrolled in the study. Clinical congestive heart failure did not occur. The left ventricular ejection fraction did not decrease significantly from the baseline level in any of the patients except for one patient treated at level 5 whose LVEF decreased to 45% after one cycle. Since this patient was transferred to another hospital, this change could not be confirmed.

### Pharmacokinetics

**Figure 2:**
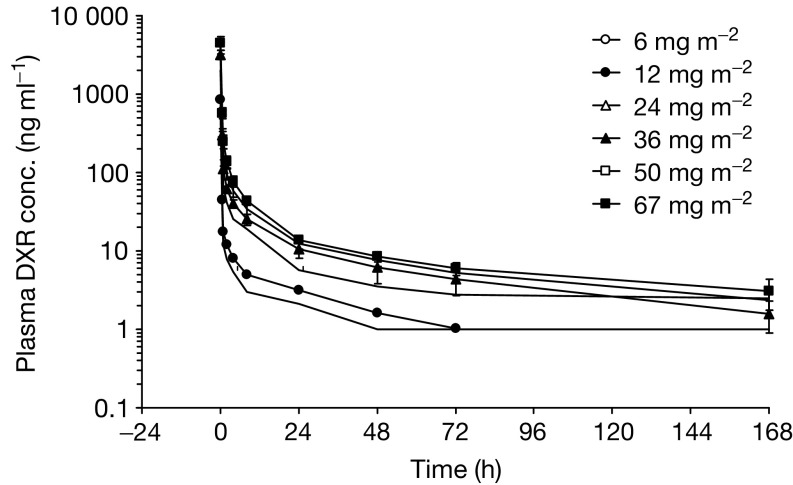
Mean plasma levels of doxorubicin following the intravenous administration of NK911 at dosages of 6, 12, 24, 36, 50, and 67 mg m^−2^ in 23 patients.

The plasma concentrations of DXR after the intravenous infusion of NK911 were determined in all of the patients enrolled in the present phase I study; the results are shown in Figure 2

**Figure 3:**
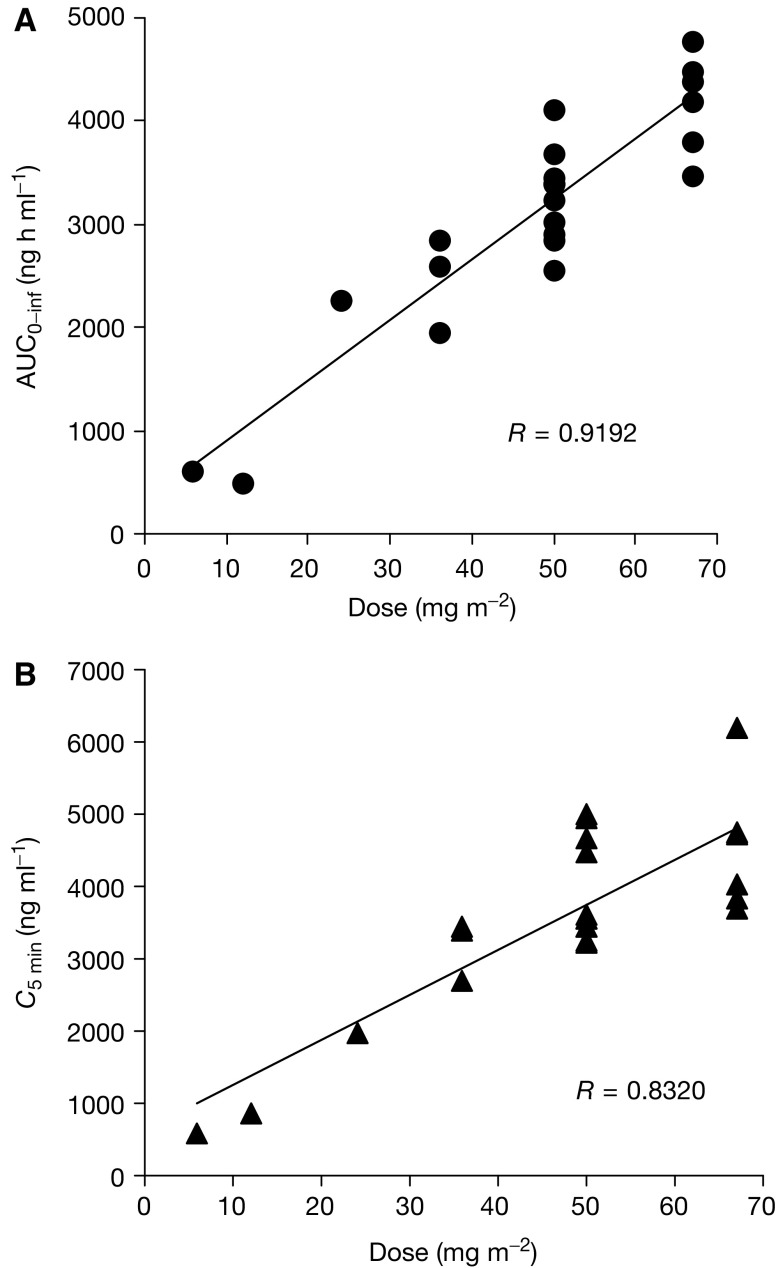
Correlations between dosage and AUC_0–inf_ (**A**) and *C*_5 min_ (**B**) after a single intravenous administration of NK911.

. The *C*_5 min_ and AUC parameters increased at doses between 6 and 67 mg m^−2^, as shown in Figure 3

. The peak plasma concentrations ranged from 586.8 ng ml^−1^ at a dose level of 6 mg m^−2^ to 6188.2 ng ml^−1^ at a dose level of 67 mg m^−2^.

The pharmacokinetic parameters are summarised in Table 4

**Table 4:** Pharmacokinetic parameters

**Dose (mg m^−2^)**	***n***	***t*_1/2*α*_ (min)**	***t*_1/2*β*_ (h)**	***T*_1/2*γ*_ (h)**	**AUC_0–inf_ (ng h ml^−1^)**	**CL_tot_ (ml min^−1^ kg^−1^)**	***V*_ss_ (l kg^−1^)**	***V*_1_ (l kg^−1^)**	**MRT_0–inf_**
NK911									
6	1	5.1	2.3	159.7	601.4	3.9	36.8	0.116	158.2
12	1	5.0	1.6	29.4	495.3	9.8	11.7	0.153	19.9
24	1	6.8	4.7	241.4	2257.7	46.1	52.3	0.163	210.9
36	3	6.6±0.5	2.8±0.0	54.8±4.8	2460.5±469.9	6.4±1.2	12.0±2.3	0.183.13±0.030	32.0±8.8
50	11	7.5±0.7	2.8±0.3	64.2±8.9	3262.7±425.2	6.7±1.1	14.9±3.6	0.167±0.035	37.0±8.2
67	6	8.1±1.1	2.9±0.5	73.6±21.4	4174.1±471.2	6.8±1.1	16.3±6.1	0.183±0.051	41.2±17.8
DXR^a^									
50	7	2.4±0.9	0.8±1.1	25.8±11.4	1620.3±1062.9	14.4±5.6	24±12	—	—
PLD^b^									
50	14	84	45.9	—	902000	0.02	0.08	—	62.7

a Cited from Mross *et al* (1998).

b Cited from Gabizon *et al* (1994).

. The initial distribution half-life (*t*_1/2*α*_) was about 5–8 min, *t*_1/2*β*_ was 1.6–4.7 h, and *t*_1/2*γ*_ was 29.4–241.4 h. The *V*1 was 0.116 –0.183 l kg^−1^. The CL_tot_ was 3.9–9.8 ml min^−1^ kg^−1^, and the *V*_ss_ was 11.7–52.3 l kg^−1^. No significant differences in these parameters were seen among patients, with the exception of patients receiving doses of 6, 12, and 24 mg m^−2^. These observations may be attributed to the fact that DXR was difficult to detect at 168 h after dosing at levels of below 24 mg m^−2^, possibly resulting in an underestimation or an overestimation of the tails of the clearance curve. The half-lives (*t*_1/2*α*_, *t*_1/2*β*_, and *t*_1/2*γ*_) were longer for NK911 than for free DXR (Mross *et al*, 1988). The AUC of NK911 was two-fold larger than that of free DXR at a dose of 50 mg m^−2^. The *V*_ss_ and CL_tot_ of NK911 were lower than those of free DXR. As expected, the parameters for NK911 were more than 100-fold lower than those previously described for doxil (Gabizon *et al*, 1994).

The cumulative urinary excretion rates of DXR and its metabolites (0–72 h) after the administration of NK911 were 7.1–16.6%, similar to those after the administration of free DXR.

### Therapeutic response

At the time of the study’s completion, eight patients (including one patient with colonic cancer and two patients with stomach cancer) had exhibited a stable disease for longer than 4 weeks (Table 5

**Table 5:** Antitumour activity

**Primary tumour**	***n***	**PR**	**SD**	**PD**
Pancreas	7	1	1	5
Colorectal	7	0	4	3
Oesophagus	3	0	1	2
Gall bladder	3	0	0	3
Stomach	2	0	2	0
Leiomyosarcoma	1	0	0	1

). A partial response was seen in one patient with metastatic pancreatic cancer who had been treated at a dosage level of 6; the size of the liver metastasis had decreased by more than 50%, compared to the baseline scan, in this patient (Figure 4A and B

**Figure 4:**
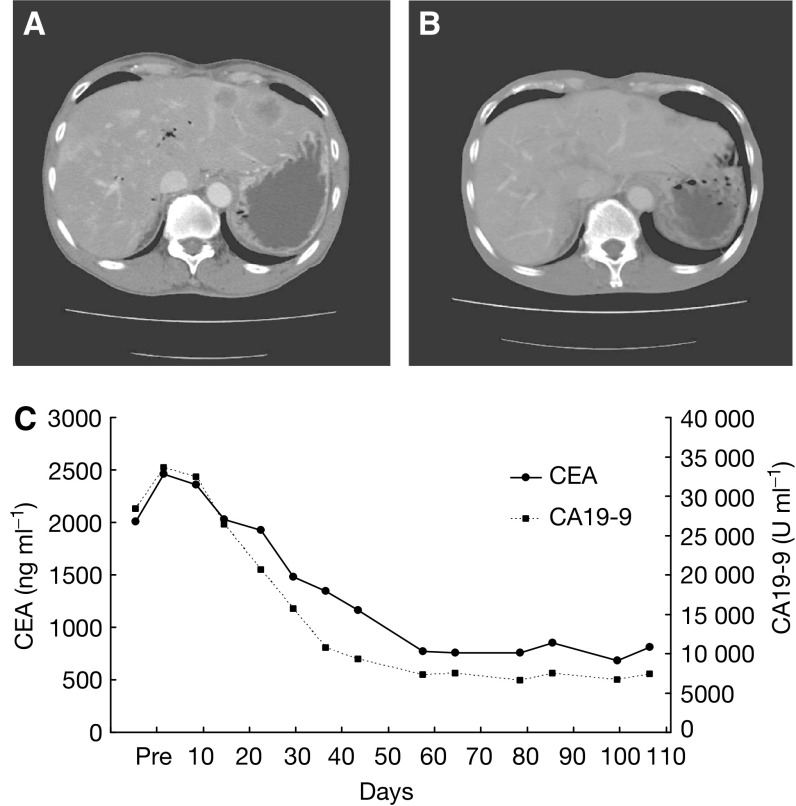
Serial CT scans of a 69-year-old male with pancreatic cancer who was treated with NK911 at an initial dosage level of 67 mg m^−2^ during the first course and at 50 mg m^−2^ after the second course. Changes in tumour marker levels are also shown. (**A**) Baseline scan showing a metastasis in the left lateral lobe. (**B**) Partial response, characterised by a more than 50% decrease in the size of the liver metastasis compared with the baseline scan. (**C**) Tumour markers, CA19-9 and CEA, decreased remarkably after treatment.

). The tumour marker (CA19-9 and CEA) levels in this patient had also decreased remarkably (Figure 4C). This patient had previously undergone gemicitabine chemotherapy. Initially, this patient received an NK911 dosage of 67 mg m^−2^. The dosage was decreased to 50 mg m^−2^ for the second course, however, because the patient experienced grade 4 neutropenia. The antitumour response was maintained even after the dosage was reduced.

## DISCUSSION

The utility of polymeric micelles in cancer chemotherapy was demonstrated in mice for the first time using DXR-incorporated polymeric micelles in the early 1990s (Yokoyama *et al*, 1990a, 1990b, 1991). The original form of micellar DXR contained two entrapped components, DXR monomers and DXR dimers, in the inner core. The DXR dimers were thought to contribute to the stabilisation of the micellar DXR conformation. However, the DXR dimers in this formulation caused freeze-dried samples of micellar DXR to become water-insoluble after prolonged storage. To improve the solubility of micellar DXR, a new polymeric micellar preparation, NK911, containing only DXR monomers was created (Nakanishi *et al*, 2001).

In this phase I study, the toxicity spectrum of NK911 resembled that of free DXR: the DLT was neutropenia, and no adverse effects appeared other than those also encountered with the use of free DXR. Regarding nonhaematological toxicities, nausea and vomiting were mild. Mucositis was also rare and mild. No infusion-related reactions, which are sometimes seen in cases of liposomal drug administration (Uziely *et al*, 1995; Muggia *et al*, 1996; Stewart *et al*, 1998; Gordon *et al*, 2001), occurred in this trial. Ultimately, this phase I study showed that the recommended dosage of NK911 (50 mg m^−2^) using a 3-week schedule was similar to the recommended dosage of free DXR (40–60 mg m^−2^). In preclinical studies using several kinds of animals, the pharmacokinetics of NK911 differed from those of free DXR (Nakanishi *et al*, 2001).

When compared with free DXR, NK911 exhibited longer half-lives (*t*_1/2*α*_, *t*_1/2*β*_, and *t*_1/2*γ*_), a lower CL_tot_, and a larger AUC; these findings suggest that the circulation of NK911 in plasma was prolonged. Furthermore, the extent of the NK911 distribution in the tumour tissue was thought to differ from that of free DXR because of the smaller *V*_ss_ of NK911 compared with that of free DXR. The *V*1 of NK911 was smaller than the volume of extracellular fluid in humans, which may lead to a smaller distribution of NK911 in tumour tissue during the early phase of chemotherapy. Although the value of *V*1 in humans after the injection of free DXR has not been reported, the *V*1 of free DXR was two-fold larger than that of NK911 in a dog model and a different distribution in the early phase was observed (data not shown); a similar tendency in humans is expected. As mentioned above, the micelle-forming ability of NK911 seems to result in different physicochemical behaviours and a specific retention in plasma, compared with DXR, because NK911 circumvents the early distribution phase. The early pharmacokinetic phase may represent a very important stage in the overall behaviour of DXR in the body (Robert *et al*, 1987). Therefore, the inherent characteristic pharmacokinetics of NK911 in human subjects seems to be useful and significant for the enhancement of clinical responses to DXR.

Concerning the release of DXR from the conjugated block copolymer, the DXR concentrations in plasma were assessed after administering a DXR-conjugated polymer in a dog model. The released DXR concentration from the polymer was estimated to be 100-fold less than that of NK911 containing the same amount of DXR-conjugated polymer in dogs. Consequently, conjugated DXR is likely to have little effect on the plasma DXR concentration after the injection of NK911 in patients. When [^14^C]DXR-conjugated polymer was intravenously administered to rats, the polymer was excreted via both urine and faeces (urine : faeces=2 : 1). In the rat urine, several kinds of fragmented polymers derived from the DXR-conjugated polymer were observed, as well as a nonfragmented polymer. In rat faeces, only the fragmented polymers were observed, and [^14^C]DXR was not seen in either urine or faeces. These results indicated that the DXR-conjugated polymer was excreted after extensive metabolism, but that the free DXR was hardly released from the polymer. Until now, the structure of the excreted high-molecular fragments could not be determined because of the difficulty in developing an analytical methodology for these molecular species. In the present clinical trial, which examined the urinary excretion of the physically trapped DXR in NK911, the excretion rate was similar to that of free DXR over a 72-h period. The present results suggest that the major route of excretion of DXR and its metabolites after NK911 injection in human subjects is likely biliary, the same as for DXR administration.

When compared with the historical data for doxil (Gabizon *et al*, 1994), the plasma AUC of NK911 was more than 100-fold lower than that of doxil, and the plasma clearance of NK911 was approximately 400-fold higher than that of doxil at a DXR equivalent dosage of 50 mg m^−2^. These results indicate that NK911 is less stable in plasma than doxil, since the DXR dimers in the micellar DXR have not been included in NK911 for the reason described earlier. Thus, doxil appears to deliver DXR to solid tumours via the EPR effect more efficiently than NK911, since doxil is more stable in the bloodstream. However, the *V*_ss_ of NK911 at a dose of 50 mg m^−2^ was about 180-fold higher than that of doxil at the same dose level. This observation suggests that the distribution of DXR in tumour tissue may be wider in the case of NK911 administration compared to that of doxil, once each formulation extravasates from the tumour vessels. Therefore, to determine whether doxil is more effective for the treatment of solid tumours than NK911, several factors must be considered, including drug pharmacokinetics, pharmacodynamics, tumour vasculature, the tumour interstitium, the efficiency of drug release from the formulation, and the distribution of free drug throughout the tumour tissue.

In conclusion, the toxicity characteristics and antitumour activity of NK911 justify its continued clinical evaluation. A phase II clinical trial of NK911 for the treatment of metastatic pancreatic cancer is ongoing.
